# A Swim in Salt Lake

**Published:** 1892-03

**Authors:** 


### A SWIM IN SALT LAKE.

A writer thus describes his experiences in a Salt Lake bath :—

During a visit to Salt Lake I " enjoyed " a swim on a summer afternoon, making the trip from town to Lake Point, the Coney Island of the region, on an excursion train in company with a number of Mormons, says a writer in Goldthwaite's Magazine. The little railroad runs through a diversified track, in which garden, farm, rocky uplift, and mud plain are oddly jumbled—the plain being spotted with tufts of pale and bristling sage brush that grows on the rocky mountain country where nothing else will. There is a bathing pavilion at Lake Point, with fresh water tanks, in which to rinse one's self after the bath, but I elected to try a swim without spectators ; so, walking southward along the shore for a mile or so, I found a place where the rounded rocks that formed the semblance of a beach, were not too numerous. It was a trifle difficult to keep a steady footing in the water, and at first I attributed this to inequalities on the bottom, but on getting where it was deeper I found that my legs had a disposition to come to the top, and it was apparent that the difficulty of wading across arose from the buoyancy that the body has in so dense a medium as this brine. When I had waded out so far that the water came up to my neck I scaled a bowlder and dived. As it is my custom to open my eyes under water, I did so as soon as I was fairly immersed. In an instant it seemed as if vitrol was poured into them. Springing to an upright position as soon as possible, I tried to get the salt out of them, but the more I rubbed the more it seemed to get in. Nature relieved the smart after awhile by pouring through the tear ducts enough of a milder solution of salt to clear the irritated cornea of the fluid, and I took pains not to let the water into my eyes again.

After that the bath was more enjoyable if only as a new experience. There was a singular and unaccustomed sense of lightness, and it was not difficult to float high out of water, either in a reclining or a sitting posture, yet a bather who is not a swimmer will fare as badly here as any where, for the head being heavier than the lower extremities, has a tendency to sink, unless one has the skill to keep it above the surface. To a swimmer there is no especial danger, unless he is choked by the brine or blinded and confused by it. To float requires less exertion than in the sea, a slight motion of the hands being sufficient to keep the body balanced evenly, for one depends less for his buoyancy on breathing than in ocean water. On striking out to swim I was surprised at a splashing noise behind me and discovered that it was made by my own feet, for I was so high out of water that they went into the air at every stroke. This lightness at one end of the body tends as I have said, to depress the other, but to one who is used to swimming this is a trifle. On emerging from the lake I found that every inch of my skin was sparkling with salt crystals, and though I rubbed and scraped they were not so easily to be got rid of. These crystals were sharp enough to create discomfort and to suggest an undue intimacy with thistles. My hair was full of them, and they even adhered to my clothing, so that a very vigorous shaking of raiment and a fresh-water bath were in order on reaching my hotel. When I told the people in town of my swim and the manner of it I was laughed at and informed that it was not the correct thing to swim except at a bathing pavilion, where one has fresh water to shower away the salt that sticks to him.

Salt Lake is by no means a saturated solution of salt, yet is five or six times as rich in salts as the ocean, and nearly as strong as the Dead sea, In summer it contains between 20 and 22 per cent. of salt, the saturation point not being reached until the salt forms a little over a third of the liquid. There are all through the great basin numerous saline lakes and ponds, but none of the size and importance of this in Utah. Not infrequently they are shallow and entirely disappear during the dryness and heats of summer, leaving to mark their sites only a stretch of some acres—or it may be miles—of clay and mud, entirely covered with salt.

